# Identification and expression of *GRAS* family genes in maize (*Zea mays* L.)

**DOI:** 10.1371/journal.pone.0185418

**Published:** 2017-09-28

**Authors:** Yuyu Guo, Hongyu Wu, Xiang Li, Qi Li, Xinyan Zhao, Xueqing Duan, Yanrong An, Wei Lv, Hailong An

**Affiliations:** State Key Laboratory of Crop Biology, College of Life Sciences, Shandong Agricultural University, Tai’an, China; Wuhan University, CHINA

## Abstract

*GRAS* transcriptional factors have diverse functions in plant growth and development, and are named after the first three transcription factors, namely, GAI (GIBBERELLIC ACID INSENSITIVE), RGA (REPRESSOR OF GAI) and SCR (SCARECROW) identified in this family. Knowledge of the *GRAS* gene family in maize remains was largely unknown, and their characterization is necessary to understand their importance in the maize life cycle. This study identified 86 *GRAS* genes in maize, and further characterized with phylogenetics, gene structural analysis, genomic loci, and expression patterns. The 86 *GRAS* genes were divided into 8 groups (SCL3, HAM, LS, SCR, DELLA, SHR, PAT1 and LISCL) by phylogenetic analysis. Most of the maize *GRAS* genes contain one exon (80.23%) and closely related members in the phylogenetic tree had similar structure and motif composition. Different motifs especially in the N-terminus might be the sources of their functional divergence. Segmental- and tandem-duplication occurred in this family leading to expansion of maize *GRAS* genes and the expression patterns of the duplicated genes in the heat map according to the published microarray data were very similar. Quantitative RT-PCR (qRT-PCR) results demonstrated that the expression level of genes in different tissues were different, suggesting their differential roles in plant growth and development. The data set expands our knowledge to understanding the function of *GRAS* genes in maize, an important crop plant in the world.

## Introduction

The *GRAS* gene family is an important plant-specific transcription factor family whose name is an acronym of the first three identified members: GIBBERELLIC ACID INSENSITIVE (GAI), REPRESSOR OF GA1 (RGA), and SCARECROW (SCR) [1]. Typically, GRAS proteins are 400–700 amino acids and exhibit some C-terminal homology. A common characteristic of all GRAS proteins is the presence of 5 carboxy-terminal motifs in the order of: leucine heptad repeat Ⅰ(LHRI), VHIID motif, leucine heptad repeat Ⅱ(LHRⅡ), PFYRE motif, and SAW motif [2–3]. Leucine heptad repeats are frequently found in bZIP transcription factors, which are important for protein-protein interactions [4]. The VHIID and PFYRE motifs are consistently found and highly conserved in GRAS proteins. The SAW motif is less conserved, but has 3 conserved pairs of amino acids found with consistent spacing: R-E, W-G, and W-W. While the function of these motifs is unknown, their highly conserved nature suggests that they are critical to GRAS protein function [5]. These conserved motifs can directly affect the function of GRAS proteins; in fact, mutations in the SAW and PFYRE motifs of SLR1 and RGA proteins result in huge phenotypic variation in *Arabidopsis* [6–7]. The order of these conserved motifs is similar across most GRAS proteins. In contrast, the N-terminus is variable, except for DELLA subfamily that contains 2 conserved motifs (named DELLA and TVHYNP) and the variable length and sequence of N-terminus seems like the major contributor for their gene-specific functions [8, 9, 10]. The GRAS family is divided into 7 subfamilies at first, DELLA, SCARECROW (SCR), LATERAL SUPPRESSOR (LS), HAIRY MERISTEM (HAM), Phytochrome A signal transduction 1 (PAT1), SHORT-ROOT (SHR) and SCARECROW-LIKE9 (SCL9) [1, 4]. Then the SCL9 was renamed as LISCL, and a new subfamily SCL3 was established and the *GRAS* gene family was divided into eight distinct branches, namely LISCL, PAT1, SCL3, DELLA, SCR, SHR, LS and HAM, based on studies on the model plants *Arabidopsis* and rice [7]. Later, the GRAS family was divided into ten subfamilies, DELLA, AtSCR, AtLAS, HAM, AtPAT1, AtSHR, AtSCL3 and LISCL, DLT, AtSCL4/7 [9]. In other studies, the *GRAS* genes can be divided into at least 13 branches [11] in *Populus*, *Arabidopsis* and rice, and 16 branches [12] in *Medicago truncatula*. So far, the *GRAS* gene family has been genome-wide explored in several plant species, including *Populus*, *Arabidopsis*, rice, Chinese cabbage, *Prunus mume*, tomato, sacred lotus, grapevine, *Isatis indigotica*, *Medicago truncatula*, Castor Beans, and pine [11, 12, 13, 14, 15, 16, 17, 18, 19, 20].

The *GRAS* gene family plays a crucial role in diverse plant growth and development processes, including gibberellin signal transduction [21, 22], phytochrome A signal transduction [23, 24], axillary meristem initiation [25], shoot meristem maintenance [26], root radial patterning [27, 28]. For example, PAT1 and SCL21 are mainly involved in phytochrome A (phyA) signal transduction pathways in *Arabidopsis* *thaliana*. Light signaling via the phyA photoreceptor controls basic plant developmental processes, such as de-etiolation and hypocotyl elongation [24]. SCARECROW-LIKE 13 (SCL13) mainly involves in phyB signal transduction pathways to control basic plant developmental processes like PAT1 and SCL21 in *Arabidopsis* *thaliana* [29]. SCARECROW-LIKE 3 (SCL3) is involved in signal transduction pathways through gibberellins (GAs) and acts as a positive regulator to integrate and maintain a functional GA pathway by attenuating the DELLA repressors in the root endodermis [30]. DELLA subfamily is a representative subfamily of GA signaling that has been analyzed in detail. Gain- or loss-of-function mutants of the DELLA genes in *Arabidopsis*, maize, wheat, rice (*Oryza sativa*) and barley show GA-insensitive dwarf or GA constitutive response phenotypes [21, 31, 32, 33]. Studies of the *Arabidopsis* ga1-3 (RGA) and the rice SLENDER RICE 1 (SLR1) have demonstrated that these DELLA proteins function in the nucleus and are degraded rapidly when the plants are treated with GA [6, 34]. The degradation of the DELLA proteins is thought to be an essential event in GA signal transduction. Mutations which lack the DELLA motif or the surrounding regions cannot be degraded by treated with GA, and show a GA-insensitive dwarf phenotype [6, 35, 36]. Previous studies showed that miR171 can regulate some *GRAS* genes. The miR171-targeted SCL transcription factors SCL6/SCL6-IV, SCLL22/SCL6-III, SCL27/SCL6-II (also known as hairy meristems [HAM] and lost meristems [LOM]) have been demonstrated to play an important role in the proliferation of meristematic cells, polar organization and chlorophyll synthesis [3, 37–41]. DELLA-regulated *POR* expression is, at least in part, mediated by miR171-targeted SCLs in light [42]. *MOC1*, a member of LS subfamily, is a key gene for controlling rice tillers, which may improve the production of crops [43].

Compared with other families of transcription factors, very few researches have explored the whole genome of the GRAS families. The identification of *GRAS* members in different species was slightly different among studies. There were 32 to 34 genes identified from *Arabidopsis* [10, 11, 44], 57 and 60 *GRAS* genes were identified in rice in two reports [10, 11]. In addition, 68 *GRAS* transcription factors were identified in *Medicago truncatula* [12]. As more species have their genome sequenced available, more GRAS proteins could be identified among them. Furthermore, the genome-wide comparisons of *GRAS* family members may also be performed among several important species for evolutionary analysis.

Maize is one of the most crops in the world and it has tremendous value for providing food, forage, pharmaceuticals, and other industrial products. To improve root growth, plant height and seed size, it is necessary to explore the GRAS family in maize. With the availability of maize genome sequences [45], it is possible for us to identify all the *GRAS* family genes in maize and find the right gene which is very necessary for production or growth.

In this study, we conducted a genome-wide analysis for all the members of GRAS family in maize. The *GRAS* genes were identified with database on the website, conducted phylogenetic relationships and analyzed their protein structures and gene structures. We discerned their locations on the chromosomes and their expression patterns as well. Then, qRT-PCR was performed to confirm the expression patterns getting from the database. The data presented here is necessary to explore systematically the gene function of the maize *GRAS* family genes.

## Results and discussion

### Genome-wide identification of *GRAS* family members in maize

It is possible to identify all *GRAS* gene family members in maize because the maize genome has been sequenced [45]. Here we identified 86 *GRAS* transcription factors (ZmGRAS1-ZmGRAS86) from the maize genome and the gene location, the number of amino acids, molecular weight, theoretical pI, were analyzed and summarized in Table 1. The length of amino acid sequences encoded by ZmGRAS varied from 111 amino acids (aa) to 734 aa, and molecular weight ranged from 12308.9 to 72083.7 kDa and the pI varied from 4.4973 to 7.7965. The average value of pI was 6.44532, suggesting that the maize GRAS proteins tended to acidic. In addition, alternative splicing was found in 18 *ZmGRAS* genes, with 2 to 4 alternative splice forms (S1 Table).

**Table 1:** Characteristics of *GRAS* genes in maize.

Gene name	Gene locus	Chromosome location	Length (aa)	Molecular weight (kDa)	PI	Subgroup
ZmGRAS1	AC198366.3	chr10: 1,890,881–1,892,401	506	55337.9	7.3345	
ZmGRAS2	AC200124.3	chr6: 62,906,676–62,907,011	111	12308.9	4.6744	
ZmGRAS3	AC204621.4	chr4: 1,274,853–1,275,830	294	31370.6	9.6636	
ZmGRAS4	AC234164.1	chr9: 24,984,785–24,987,244	630	66847	6.9353	
ZmGRAS5	GRMZM2G001426	chr3: 69,955,114–69,957,195	475	50563	5.7858	
ZmGRAS6	GRMZM2G011947	chr6:55,555,919–55,556,867	304	32193.3	6.1322	
ZmGRAS7	GRMZM2G013016	chr3: 166,194,240–166,195,917	538	57765.5	6.5057	
ZmGRAS8	GRMZM2G015080	chr2: 150,778,730–150,782,551	678	72083.7	6.6531	
ZmGRAS9	GRMZM2G018254	chr4: 513,060–515,231	648	71808	6.0844	
ZmGRAS10	GRMZM2G019060	chr2: 209,040,871–209,042,963	586	63755.6	5.498	
ZmGRAS11	GRMZM2G023872	chr8: 72,494,685–72,496,501	508	56088.1	6.3404	
ZmGRAS12	GRMZM2G024973	chr5: 11,793,473–11,795,945	625	65742.6	5.0091	D9
ZmGRAS13	GRMZM2G028039	chr9: 149,430,555–149,437,059	545	60793.6	6.2568	
ZmGRAS14	GRMZM2G028438	chr4: 156,049,665–156,051,935	607	63032.1	8.5031	
ZmGRAS15	GRMZM2G028608	chr2: 46,590,168–46,592,051	607	66678.5	6.4672	
ZmGRAS16	GRMZM2G037286	chr6: 155,175,171–155,176,649	489	51240.9	6.1941	
ZmGRAS17	GRMZM2G037792	chr10: 138,720,419–138,723,128	718	75350.4	6.094	
ZmGRAS18	GRMZM2G049159	chr1: 40,143,545–40,147,542	734	82633.5	6.5233	
ZmGRAS19	GRMZM2G051785	chr9: 27,346,189–27,348,215	507	53502	5.9579	
ZmGRAS20	GRMZM2G055263	chr1: 37,809,278–37,810,975	564	59835.2	4.9556	
ZmGRAS21	GRMZM2G060265	chr1: 1,953,455–1,955,531	481	51254.8	6.5752	
ZmGRAS22	GRMZM2G070371	chr2: 231,610,740–231,612,552	599	67026.7	6.5602	
ZmGRAS23	GRMZM2G073779	chr4: 836,061–838,338	686	76470.4	4.8064	
ZmGRAS24	GRMZM2G073805	chr4: 832,522–834,779	666	73486.5	5.5435	
ZmGRAS25	GRMZM2G073823	chr4: 829,955–831,938	642	71250.6	7.1461	
ZmGRAS26	GRMZM2G079470	chr1: 88,488,422–88,491,040	645	67236.9	6.4735	
ZmGRAS27	GRMZM2G082387	chr3: 150,869,807–150,871,966	447	48471	6.8274	
ZmGRAS28	GRMZM2G089636	chr6: 38,422,200–38,424,095	626	71576.2	6.0342	
ZmGRAS29	GRMZM2G089662	chr6: 38,410,832–38,413,066	726	82524.8	7.9209	
ZmGRAS30	GRMZM2G089782	chr3: 124,035,075–124,037,072	345	37232.6	7.7965	
ZmGRAS31	GRMZM2G091656	chr4: 213,905,717–213,907,179	473	51700.4	4.4973	
ZmGRAS32	GRMZM2G097456	chr7: 162,247,332–162,249,303	456	49478.5	5.398	
ZmGRAS33	GRMZM2G098517	chr7: 161,325,637–161,329,188	558	60991.6	6.037	
ZmGRAS34	GRMZM2G098784	chr9: 140,092,351–140,096,097	734	82407.3	6.3035	
ZmGRAS35	GRMZM2G098800	chr4: 153,223,584–153,226,721	479	51399.2	8.136	
ZmGRAS36	GRMZM2G104342	chr5: 9,827,131–9,829,504	569	61233.7	4.8845	
ZmGRAS37	GRMZM2G106336	chr4: 2,654,200–2,655,918	569	64161.6	5.6074	
ZmGRAS38	GRMZM2G106356	chr4: 2,656,855–2,659,044	629	71509.2	6.1865	
ZmGRAS39	GRMZM2G106548	chr7: 158,406,894–158,408,665	452	48411.2	6.4457	
ZmGRAS40	GRMZM2G109869	chr2: 15,351,727–15,354,059	623	65571	6.5741	
ZmGRAS41	GRMZM2G110067	chr1: 71,030,279–71,032,027	526	54677.3	7.1968	
ZmGRAS42	GRMZM2G110579	chr5: 192,899,281–192,902,025	595	62880.7	5.9948	
ZmGRAS43	GRMZM2G114680	chr9: 103,353,117–103,362,197	467	49942.9	9.5395	
ZmGRAS44	GRMZM2G116638	chr1: 137,434,183–137,440,188	542	59914.4	6.1403	
ZmGRAS45	GRMZM2G117949	chr9:117,530,782–117,531,764	118	12754.8	7.2349	
ZmGRAS46	GRMZM2G125501	chr2: 209,545,748–209,547,741	456	49134.1	5.2625	
ZmGRAS47	GRMZM2G129154	chr8: 158,560,400–158,562,023	449	48889.6	6.7131	
ZmGRAS48	GRMZM2G131516	chr4: 185,481,408–185,484,703	140	15229.2	5.6514	SCR
ZmGRAS49	GRMZM2G132794	chr1: 77,494,290–77,496,510	630	67547.3	6.6294	
ZmGRAS50	GRMZM2G133169	chr1: 270,571,160–270,573,052	551	59842.1	5.0325	
ZmGRAS51	GRMZM2G140085	chr4: 187,217,605–187,218,942	369	39334.6	7.342	
ZmGRAS52	GRMZM2G140094	chr4: 187,219,736–187,222,248	487	51456.3	6.6281	
ZmGRAS53	GRMZM2G143433	chr5: 120,520,869–120,522,119	416	43484.9	5.5444	
ZmGRAS54	GRMZM2G144744	chr1: 266,160,101–266,163,168	630	66028.9	4.7928	D8
ZmGRAS55	GRMZM2G146018	chr3: 142,759,880–142,761,574	492	52614.5	4.9922	
ZmGRAS56	GRMZM2G153333	chr6: 148,102,723–148,106,458	561	62826.6	4.6747	
ZmGRAS57	GRMZM2G157679	chr3: 180,798,590–180,801,759	809	89139.9	6.4786	
ZmGRAS58	GRMZM2G159475	chr3: 125,733,039–125,735,799	710	78391.5	5.1754	
ZmGRAS59	GRMZM2G163427	chr4: 842,617–845,669	765	84654.3	5.1643	
ZmGRAS60	GRMZM2G169636	chr7: 165,196,140–165,197,777	545	57959.5	7.0312	
ZmGRAS61	GRMZM2G172657	chr7: 161,941,687–161,944,142	592	63904.6	5.9479	
ZmGRAS62	GRMZM2G173429	chr10: 135,825,173–135,827,459	623	65303.7	6.7864	
ZmGRAS63	GRMZM2G176537	chr10: 117,338,432–117,340,217	494	52101.9	5.5598	
ZmGRAS64	GRMZM2G179325	chr1: 155,690,714–155,694,188	721	79879	4.8103	
ZmGRAS65	GRMZM2G313078	chr6: 156,946,311–156,947,989	426	45514.7	6.5092	
ZmGRAS66	GRMZM2G317287	chr10: 1,886,674–1,888,997	606	66692.2	8.0803	
ZmGRAS67	GRMZM2G335814	chr1: 248,734,599–248,737,101	732	82795.3	6.547	
ZmGRAS68	GRMZM2G342217	chr4: 21,604,764–21,607,924	771	82103.3	6.6613	
ZmGRAS69	GRMZM2G346706	chr8: 133,612,993–133,614,885	627	70612.1	7.267	
ZmGRAS70	GRMZM2G348780	chr1: 161,047,325–161,049,647	338	38237.4	9.306	
ZmGRAS71	GRMZM2G359304	chr9: 123,178,843–123,180,450	535	55755.5	6.4503	
ZmGRAS72	GRMZM2G368909	chr1: 293,715,271–293,717,691	645	72646.3	6.9815	
ZmGRAS73	GRMZM2G386362	chr2:4,624,290–4,626,096	354	36607.9	6.2392	
ZmGRAS74	GRMZM2G408012	chr5: 181,911,451–181,912,595	251	28281.4	10.717	
ZmGRAS75	GRMZM2G418899	chr7: 162,852,139–162,862,787	431	47773.6	5.7168	
ZmGRAS76	GRMZM2G420280	chr2: 51,272,702–51,274,207	498	53733.3	8.6598	
ZmGRAS77	GRMZM2G425366	chr2: 219,524,850–219,526,665	541	60489.3	5.2866	
ZmGRAS78	GRMZM2G431309	chr2: 208,500,828–208,504,903	554	60835.6	6.23	
ZmGRAS79	GRMZM5G821439	chr2: 204,949,825–204,954,417	570	64511.1	6.3587	
ZmGRAS80	GRMZM5G825321	chr2: 19,769,962–19,772,850	485	52611.8	7.2682	
ZmGRAS81	GRMZM5G826526	chr3: 214,641,511–214,642,665	384	40366.2	7.5029	
ZmGRAS82	GRMZM5G868355	chr4: 839,965–841,260	381	42723.6	6.5888	
ZmGRAS83	GRMZM5G874545	chr3: 232,137,122–232,158,937	342	37832.3	6.5808	
ZmGRAS84	GRMZM5G885274	chr5: 205,139,992–205,141,687	545	60793.6	6.2568	
ZmGRAS85	GRMZM5G889326	chr6: 137,480,065–137,481,525	486	52641.4	7.2535	
ZmGRAS86	GRMZM5G895672	chr4: 827,079–829,016	645	72101.2	6.8419	

However, inconsistent with our results, there were 104 and 112 GRAS genes of maize in the PlantTFDB website (http://planttfdb.cbi.pku.edu.cn/family.php?sp=Zma&fam=GRAS) and PlnTFDB website (http://plntfdb.bio.uni-potsdam.de/v3.0/fam_mem.php?family_id=GRAS&sp_id=ZMA), respectively (S2 Table). The number of the GRAS genes in the two websites was greater than our results. After analysis carefully, we found that the 104 GRAS genes of maize in the PlantTFDB website were very different from the 112 GRAS genes in the PlnTFDB website. Genes written in the red words were the different genes between the two websites in S2 Table. The 104 GRAS genes in the PlantTFDB website included alternative splicing genes. If the “genes” containing splice variants were considered one gene, there remained only 86 GRAS genes which was consistent with our result above. For example, the gene “GRMZM2G015080”in the PlantTFDB website existed two transcripts which were considered to be two genes, but only one gene, actually. In addition to alternative splicing, most of the GRAS genes in the PlnTFDB website could not be found in the MaizeGDB website (http://www.maizegdb.org/gene_center/gene) probably owning to the low version. We downloaded the GRAS protein sequences of *Arabidopsis thaliana*, *Medicago truncatula*, *Oryza sativa and Sorghum bicolor* from the PlantTFDB website.

Very few reports have been published on *Zea mays* GRAS proteins, to our knowledge, three of these genes were previously described, such as, D9 (*ZmGRAS*12) [45, 46], D8 [*ZmGRAS54*) [45, 47, 48] and SCR (*ZmGRAS48*) [49, 50](Table 1). The relatively high number of *GRAS* genes in maize may be due to the maize genome experiencing tandem and large-scale segmental duplications [51].

### Phylogenetic analysis of *GRAS* genes

**Fig 1:**
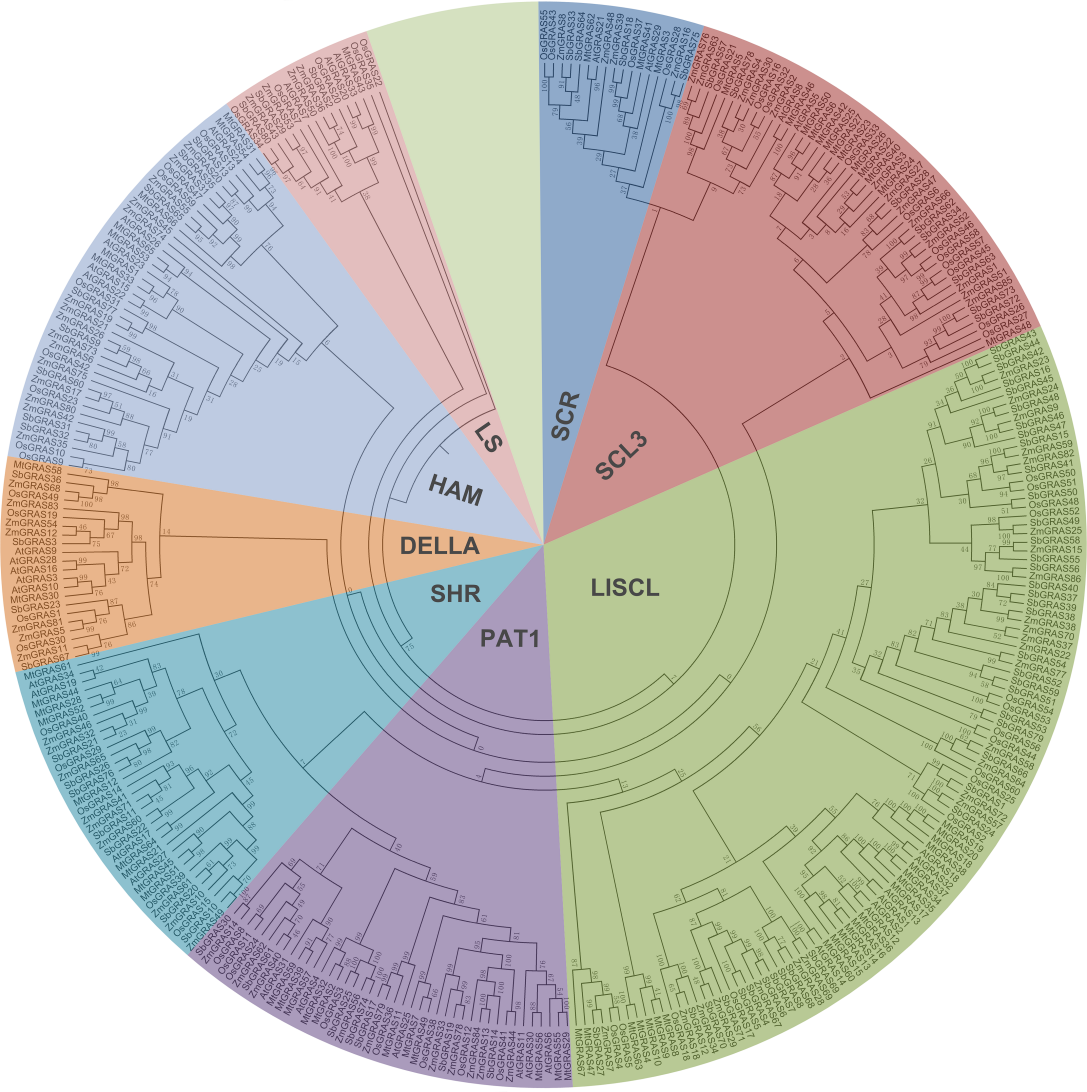
Phylogenetic relationships of GRAS proteins from *Zea mays*, *Arabidopsis thaliana*, *Oryza sativa*, *Sorghum bicolor* and *Medicago truncatula*. The proteins are clustered into 8 subgroups, signed in 8 different colors, representing subfamilies of SCR (blue), SCL3 (red), LISCL (green), PAT1 (purple), SHR (light blue), DELLA (orange), HAM (light purple), LS (pink). Gene ID of *GRAS* gene family members from *Arabidopsis thaliana*, *Oryza sativa*, *Sorghum bicolor* and *Medicago truncatula* were listed in the supporting information (S3 Table).

To study evolutionary relationships between *GRAS* transcription factors, the sequences of *Arabidopsis*, *Medicago truncatula*, *Oryza sativa* and *Sorghum bicolor* were downloaded for alignment and were used to conduct phylogenetic tree by PHYLIP (Version 3.695) using Neighbor-Joining method [52, 53]. The 86 maize GRAS proteins comprise 8 subfamilies (SCL3, HAM, LS, SCR, DELLA, SHR, PAT1 and LISCL) by clade support values, tree topology and *Arabidopsis* classification (Fig 1) [7]. Each of the SCR and LS subfamily contained only four maize *GRAS* genes and was the relatively small subfamily of the whole subfamilies. The number of maize *GRAS* genes in SHR, PAT1, DELLA, HAM, SCL3 was very similar about ten, while the LISCL subfamily was very different from the above subfamilies that contained the largest number of maize *GRAS* genes and this branch was also the largest branch in Fig 1, contained 106 members from *Zea mays*, *Arabidopsis* thaliana, Oryza sativa, *Sorghum bicolor* and *Medicago truncatula*. In this family, *GRAS* genes of eudicot plants *Arabidopsis* and *Medicago truncatula* didn’t clustered together with others tightly.

### ZmGRAS protein sequence alignments and conserved motifs

**Fig 2:**
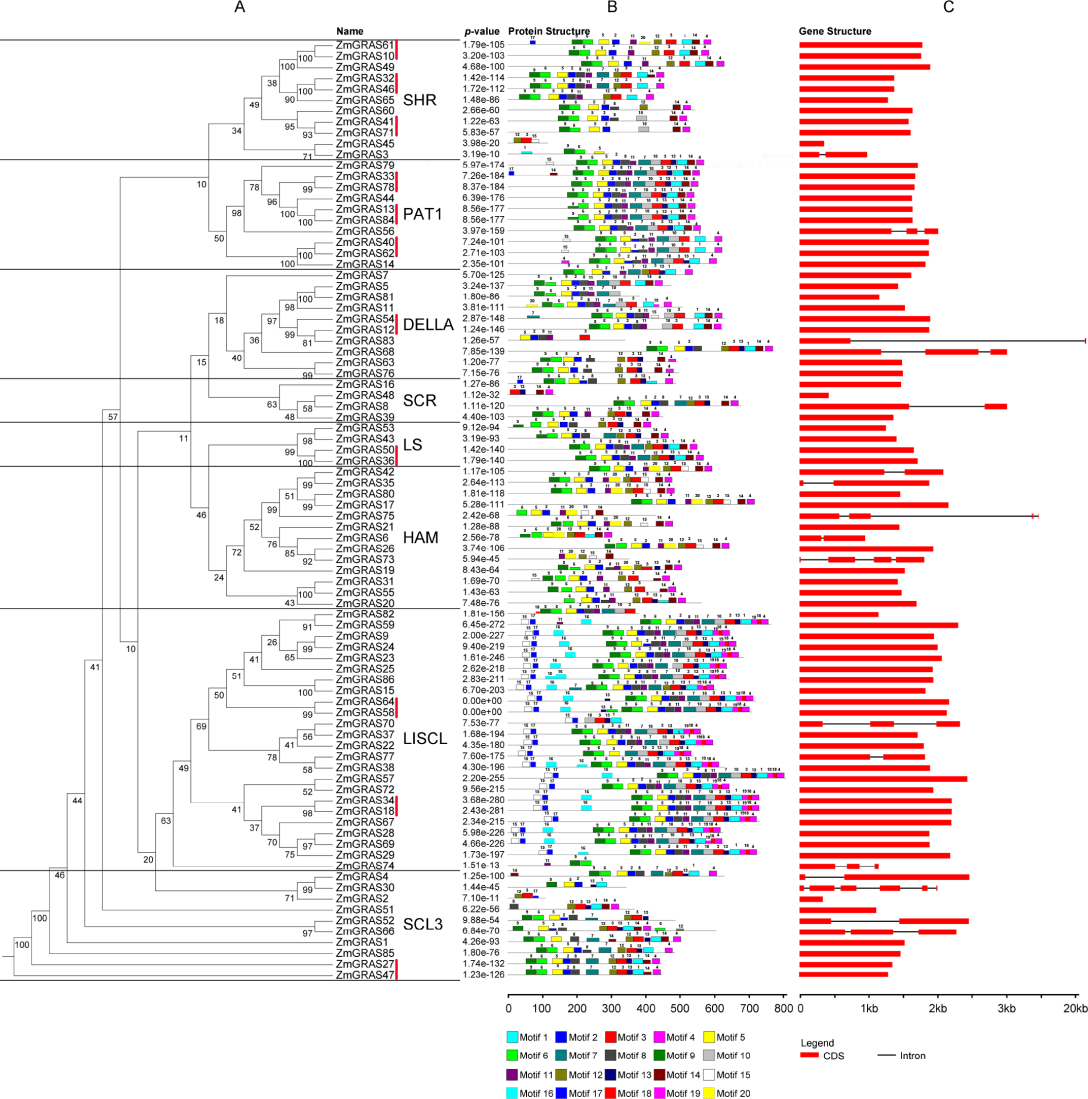
Distribution of conserved motif of GRAS proteins in *Zea mays*. (A) The phylogenetic tree was constructed by PHYLIP using NJ method. The genes marked by the red lines were duplicated gene pairs mentioned in the following paragraph. (B) The motif sizes are indicated at the bottom of the figure. Different motifs are indicated by different colors numbered from motif1-20, and the combined P-values are shown on the left side of the figure. The same color in different proteins refers to the same motif. The structural features of the 20 motifs were listed in S4 Table. (C) The structures of the 86 putative maize *GRAS* genes. The exons and introns are represented by red boxes and black lines.

In *Arabidopsis*, GRAS proteins have 5 conserved domains in the C terminus, named as LHR I, VHIID, LHR II, PFYRE and SAW [2–3]. To identify conserved domains, we performed an alignment within ZmGRAS protein sequences using Clustal X, (Version 2.0) [54]. Multiple sequence alignments of the 86 predicted ZmGRAS proteins led to the discovery of five conserved C-terminal GRAS domains mentioned above, which were similar to *Arabidopsis* GRAS proteins (S1 Fig). The conserved motifs for each GRAS protein were also identified using MEME (http://meme.sdsc.edu/meme/intron.html) (Fig 2B; S4 Table). A total of 20 conserved motifs were identified (named Motif1-20) and detailed information for each motif was listed in S4 Table. Motifs (Motif1, 2, 3, 4, 5, 6, 9 and 11) were widely distributed in most maize C-terminus of GRAS proteins, while the N-terminus contained various motifs, such as motif15, 16, 17, which was consistent with the previous conclusions that C-terminal region of the GRAS proteins was more conserved than the N-terminal region [2]. Although the motifs of the N-terminal regions of the GRAS genes were variable, it increased functional diversity and the complexity of biological networks of the GRAS genes [9, 10]. Genes among different subfamilies had divergent motifs in the N-terminal regions, but most of the GRAS proteins in the same subfamily had similar motifs. For example, In LISCL subfamily, there are three specific motifs (motif15, motif16, and motif17) in its C-terminus (Fig 2B; S2 Fig). The results were consistent with previous study that the pattern of protein disorder could be more conserved through evolution than the amino acid sequence in the N-terminus [55].

### Structural organization of *ZmGRAS* genes and chromosomal localization

The overall pattern of intron positions can affect phylogenetic relationships when analyzing gene family evolution [56]. To evaluate the diversity of the *GRAS* genes, we analyzed the structure of each maize *GRAS* gene. The result showed that 69 (80.23%) *ZmGRAS* genes with non-intron and only 17 (19.77%) genes with 1–5 intron (Fig 2C). There were nine genes with one intron, five genes with two introns, two genes with three introns, and only one gene with five introns. In addition, most *GRAS* gene members of the same branch generally showed similar exon-intron structures.

To investigate the chromosomal distribution of the GRAS family in maize, the physical location information of the maize *GRAS* genes on chromosomes according to the phytozome database (http://phytozome.jgi.doe.gov/, v3) was used to draw the map. The 86 *GRAS* genes demonstrated a nonrandom distribution. More than one third of the *GRAS* transcription factors were found on two chromosomes: chromosome 4 (n = 17, 19.77%) and chromosome 1 (n = 13, 15.12%), and only 3 (3.49%) on chromosome 8. *ZmGRAS* genes were not found on the short arms of chromosome 6, 7 and 8, and only one *GRAS* gene (*ZmGRAS5*) was found on the short arm of chromosome 3. Eleven *ZmGRAS* genes were clustered at the end of the short arm of chromosome 4.

**Fig 3:**
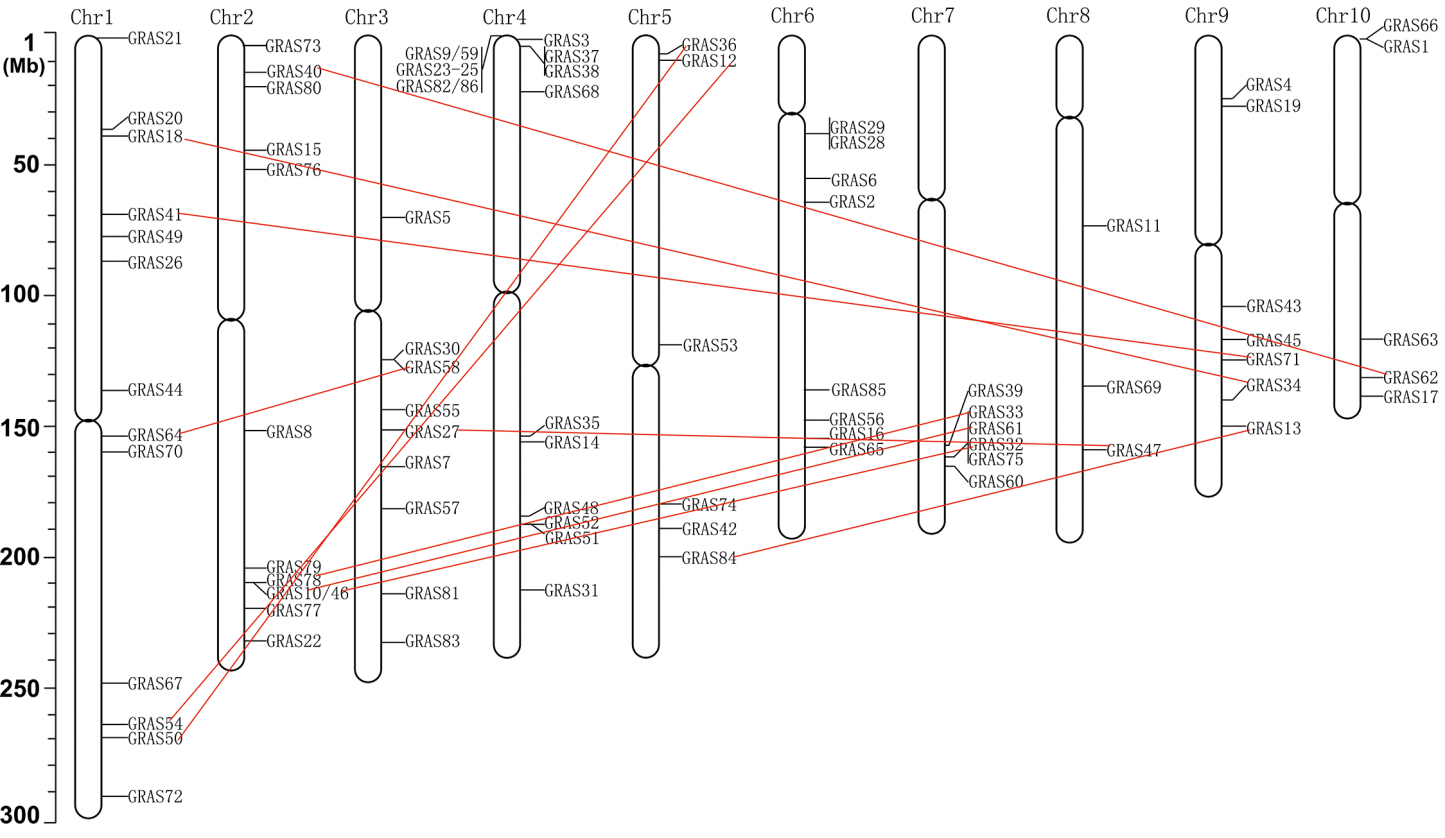
The chromosomal mapping analysis of the *GRAS* gene family in maize and gene duplication analysis. The chromosome number is indicated at the top of each chromosome. Eleven pairs of paralogues are indicated by the red line.

Duplication events are of interest in across many taxa, and maize originates from an ancient allotetraploid event and has undergone several rounds of polyploidy [45, 57]. We identified eleven duplicated genes with highly amino acid sequence and structure similarities, and all of them contain only one exon. These duplicated genes belonged to six groups with six, six, four, two, two, two genes in SHR, PAT1, LISCL, DELLA, SCL3, LS subfamily, respectively, each of the duplicated genes contained two genes with very close genetic relationship (Fig 2; Fig 3). Five of these duplicated genes were distributed on chromosome 1 and none of them on chromosome 4, and 6. In addition, two pairs duplicated genes on chromosome 2 and 7 (*ZmGRAS10/ZmGRAS61*, *ZmGRAS32/ZmGRAS46*) belonging to the same SHR subfamily. These results suggested that segmental duplication has played a role in subfamily’s origin [40].

### Expression of *ZmGRAS* genes in different tissues and developmental stages

**Fig 4:**
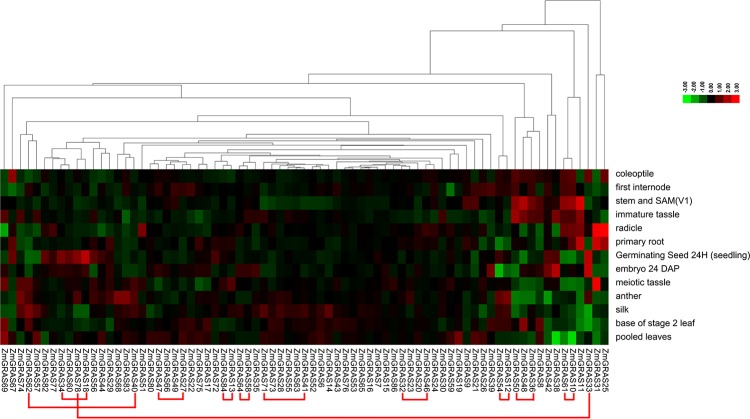
The expression of *GRAS* gene family members in different tissues of maize. In the heat map, columns represent genes, while rows represent different tissues, including germinating seed 24 H (seedling), coleoptile, radicle, stem and SAM (V1), first internode, immature tassel, meiotic tassel, anthers, primary root, pooled leaves, silks, base of stage 2 leaf (adult leaf), and embryo 24 DAP. The color changes from green to red represent the relative low or high expression in leaves respectively. The red lines represent eleven duplicated genes pairs.

*GRAS* transcription factors have important roles in plant growth and development, such as cell maintenance and proliferation, axillary shoot meristem formation, root radial pattering, and male gametogenesis. Genes expressed high in particular tissues may play essential roles in the development of the tissues. The expression of *GRAS* genes in different tissues and different developmental stages were analyzed using published microarray data (Maize eFP Browser, http://bar.utoronto.ca/efp_maize/cgi-bin/efpWeb.cgi). The expression data included 13 different tissues, including germinating seed 24 H (seedling), coleoptile, radicle, stem and SAM (V1), first internode, immature tassel, meiotic tassel, anthers, primary root, pooled leaves, silks, base of stage 2 leaf (adult leaf), and embryo 24 DAP. The expression patterns of 75 genes were found in this database (Fig 4).

In Fig 4, partial genes in a branch with similar expression patterns in different tissues were laid in the same branch in Fig 2, for example, the nine pair duplicated genes (81.82%, *ZmGRAS10/61*, *ZmGRAS12/54*, *ZmGRAS13/84*, *ZmGRAS18/34*, *ZmGRAS27/47*, *ZmGRAS36/50*, *ZmGRAS41/71*, *ZmGRAS46/32*, *ZmGRAS58/64*) in which genes within the same branch had similar expression patterns (Fig 2; Fig 4), but the duplicated gene pair *ZmGRAS40/62* belonging to the PAT1 subfamily had different expression pattern (Fig 4). We analyzed the regulator elements of the 2000bp promoter region of the gene *ZmGRAS40* and *ZmGRAS62* using plantcare (http://bioinformatics.psb.ugent.be/webtools/plantcare/html/) and New PLACE (https://sogo.dna.affrc.go.jp/cgi-bin/sogo.cgi?lang=en&pj=640&ac-tion=page&page=newplace) online websites and found that the regulator elements in front of the two promoters were not the same (S5 Table). For example, in the 2000bp promoter region of gene *ZmGRAS62* there were four “MYBPLANT motif” which couldn’t be found in the 2000bp promoter of gene ZmGRAS40, the “MYBPLANT motif” was distributed in four positions upstream of the start codon, respectively, “-387bp”, “-495bp”, “-1489bp”, “-1662bp”which was plant MYB binding site according to the New PLACE website. The AmMYB308 and AmMYB330 transcription factors from Antirrhinum regulated phenylpropanoid and lignin biosynthesis in tobacco [58]. The Lignin was one of the components of the cell wall and filled with the cellulose framework to enhance the mechanical strength of the plant, which is conducive to the organization of water transport, and affect the growth and development of organs including leaf and stem. Additionally, in the promoter of the gene *ZmGRAS62*, there existed GA-responsive elements (GARE2OSREP1 and GAREAT) which didn’t existed in the promoter of the gene *ZmGRAS40* according to the New PLACE website [59, 60]. Gibberellins (GAs) were phytohormones that regulate various aspects of plant development, including germination dormancy, leaf morphogenesis and shoot and root growth, etc [61]. We speculated that different expression level between the gene *ZmGRAS62* and *ZmGRAS40* at the stage of first internode (V5), base of stage 2 leaf (V5) and pooled leave possibly owning to the above reason. But further experiments were needed to verify this hypothesis. Additionally, partial genes with similar expression patterns belonged to different branch. For example, ZmGRAS60 and ZmGRAS34 had similar expression patterns, but they were divided into SHR subfamily and LISCL subfamily, respectively.

**Fig 5:**
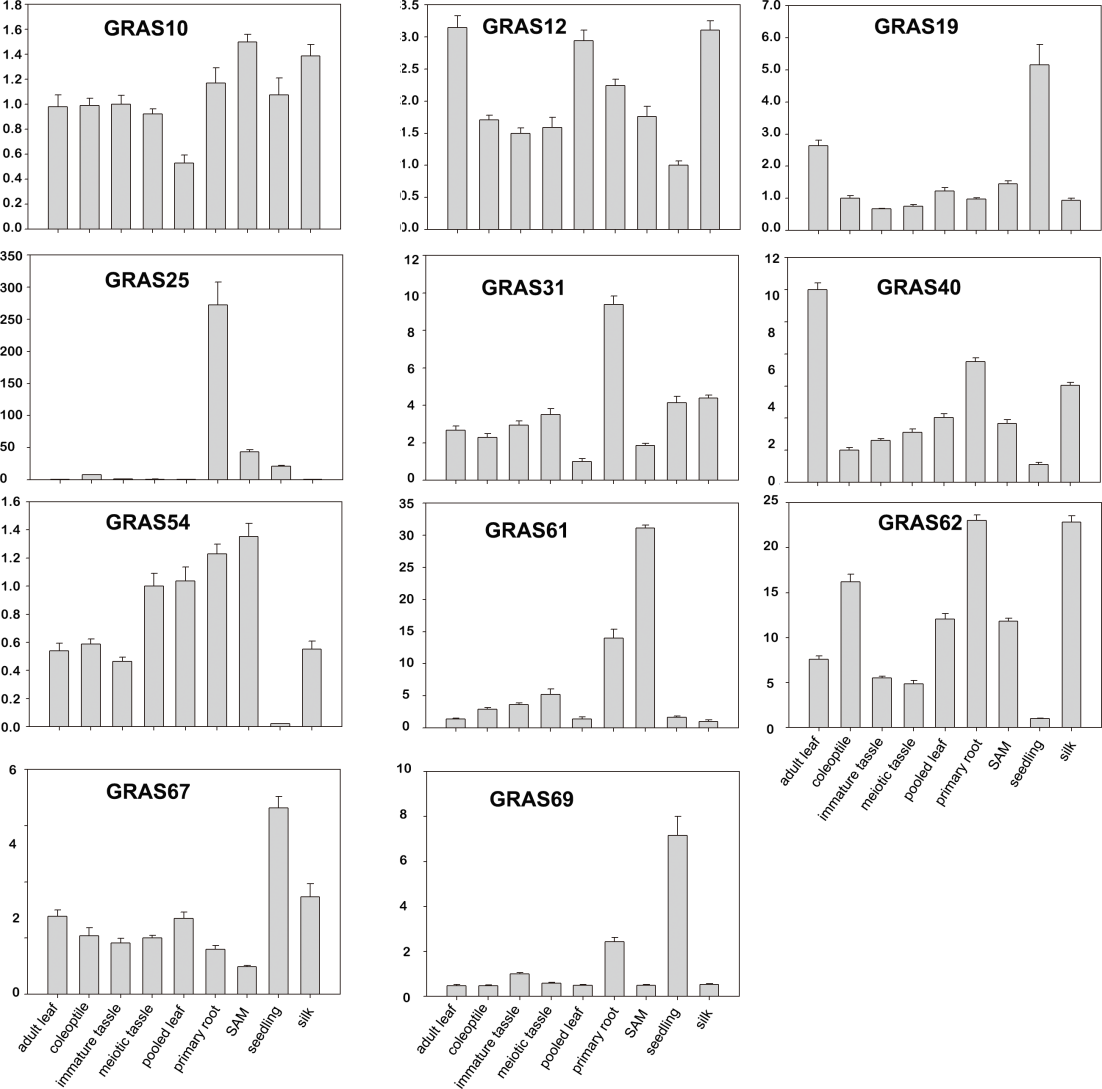
Expression patterns in 11 *GRAS* genes by real-time quantitative RT-PCR analysis. cDNAs from nine different tissues of eight developmental stages were used to detect the expression of eleven *GRAS* genes in which *ZmGRAS40*, *ZmGRAS54* and *ZmGRAS62* have multiple splice variants. The quantitative primers for *ZmGRAS40*, *ZmGRAS62* were used to test all of the variants, and the quantitative primers for the gene *ZmGRAS54* were used to test the main two transcripts (GRMZM2G144744-T01 and GRMZM2G144744-T02). The relative expression levels which were normalized to ACTIN were determined by the comparative CT method (2^−ΔΔCT ^) [62] Three biological replicates were conducted for each experiment.

Real-time quantitative RT-PCR (qPCR) was used to validate the microarray data. Eleven genes were selected to confirm expression patterns in primary root, pooled leaf, coleoptile, SAM, adult leaf, silk, seedling, meiotic tassel and immature tassel. As shown in Fig 5, 6 genes (except *ZmGRAS69*, *ZmGRAS40*, *ZmGRAS19*, *ZmGRAS12 and ZmGRAS62*) had similar expression patterns between the qPCR data and microarray data. For example, *ZmGRAS67* had higher expression in seedling, and *ZmGRAS10*, *ZmGRAS61* were mainly expressed in SAM. However, *ZmGRAS69* had high expression in seedlings in the qPCR, in contrast to the pooled leaf microarray data. These conflicting results may result from the different plant materials, different growth conditions, and different experimental conditions. These results suggest that some *GRAS* genes with different expression levels in different organs might play key roles in plant development. Several *GRAS* genes may also have unique functions during specific developmental stages.

## Conclusion

86 putative *GRAS* family genes were identified from maize via sequence comparison between maize, *Arabidopsis*, rice, *Medicago truncatula* and *Sorghum bicolor*. Only a few genes from this transcription factor family have been previously characterized in detail in maize. Thus, this work is the first comprehensive and systematic analysis of GRAS transcription factors in maize. The number of the maize *GRAS* family genes is larger than other plants, suggesting that they might encounter gene segmental-duplication and tandem-duplication during the evolution. Due to the fact that all of the maize GRAS genes expressed differentially, the genes possibly encountered sub-functionalization or neo-functionalization. So, it’s reasonable to explore each gene for its specific role in maize growth and development, to support the current work on maize molecular breeding.

## Materials and methods

### Database search for *GRAS* genes in maize

The whole protein sequences of *Zea mays* were downloaded from the MaizeGDB (http://www.maizegdb.org/, v3). The protein sequences for 34 *Arabidopsis GRAS* genes were downloaded from plant TFDB (http://planttfdb.cbi.pku.edu.cn/). HMMER 3.0 software was obtained from the HMMER website (http://hmmer.janelia.org/) and was employed in searching for GRAS proteins in the entire protein dataset of *Zea mays* with a cut-off E-value of 1e^-5^ using PF03514.11 which was the newest HMM model for the GRAS transcription factor family downloaded from the Pfam database (http://pfam.xfam.org/) [63, 64] as a query. Genes were classified according to the distance homology with *Arabidopsis* and rice genes [7].

### Phylogenetic analysis of maize GRAS proteins

All GRAS protein sequences of *Arabidopsis*, rice, *Medicago truncatula*, and *Sorghum bicolor* were downloaded from plant TFDB (http://planttfdb.cbi.pku.edu.cn/). Then, together with the 86 maize GRAS proteins, the multiple sequence alignment was performed using Clustal X, (Version 2.0) [54]. The aligned sequences were then subjected to phylogenetic analysis by Neighbor Joining (NJ) method using PHYLIP (Version 3.695) with 1000 bootstrap replicates [52, 53].

### Chromosomal location of maize *GRAS* genes

All *GRAS* genes were mapped to maize chromosomes based on information available at the Phytozome website (http://phytozome.jgi.doe.gov/, v3). The map was drafted using photoshop CS3 based on chromosome size.

### Analysis and distribution of conserved motifs and exon-intron structures

The exon-intron organization of *GRAS* genes was determined by the online GSDS 2.0 tools (Gene Structure Display Server) [65] based on the CDS sequence and corresponding genomic sequences which were obtained from the website (https://phytozome.jgi.doe.gov/pz/portal.html). Multiple EM for Motif Elicitation (MEME, http://meme-suite.org/) [66] was used to search for possible conserved motifs in the complete amino acid sequences of maize GRAS proteins using the default settings.

### Gene duplication analysis of maize *GRAS* genes

Gene duplication events of *GRAS* genes in maize B73 were investigated. We defined the gene duplication using the following criteria: 1) the alignment of whole protein length covered >80% of the longest gene, 2) the aligned region had an identity >80% and 3) only one duplication event was counted for tightly linked genes. The duplicated gene pairs were connected by red lines using photoshop CS3.

### Expression patterns of *GRAS* family in maize

The data of expression patterns of *GRAS* family genes in maize was found in Maize eFP Browser (http://bar.utoronto.ca/efp_maize/cgi-bin/efpWeb.cgi). The expression patterns of different genes were searched by primary gene ID. The expression level of different tissues was put in a table, then analyzed using Cluster (v3.0) [67] and Java Treeview (v1.1.6).

### Plant materials and growth conditions

Maize seeds (*Zea mays* L. cvB73) were grown in soil under greenhouse conditions at 25°C/22°C (day/night) with a photoperiod of 16/8 h (day/night) for 2 weeks.

### RNA isolation and real-time quantitative RT-PCR expression analysis

Primary root and pooled leaf were sampled when the first leaf is fully extended (V1), coleoptile was sampled 6 days after sowing (6 DAS), SAM was sampled when the three leaves were fully extended(V3), adult leaf was sampled when the seven leaves were extended(V5), silk was sampled when the silks emerge from the husk(R1), seedling was sampled from 24 h after imbibition, meiotic tassel was sampled when the eighteen leave were extended(V18), immature tassel was sampled when the thirteen leave were extended(V13) [68, 69], these nine different tissue materials were collected and stored for RNA isolation. Total RNA was extracted using Trizol (Invitrogen, Carlsbad, CA, USA). All the primers for qPCR were designed using QuantPrimer (http://quantprime.mpimp-golm.mpg.de/) (S6 Table). We selected the best pair of primers for qRT-PCR, the specificity of primers was tested by melting curve. A single peak indicates that the amplification product is specific and the corresponding PCR results were used for data analysis. Reverse transcription was performed with 5 μg total RNA as the template by using the TransScript® II One-Step gDNA Removal and cDNA Synthesis SuperMix (TRANSGEN BIOTECH, AH311). Quantitative RT-PCR (qRT-PCR) was carried out on the Bio-RAD CFX96 using the Real SYBR Mixture (CWBIO, CW0760). The results were analyzed with the Bio-RAD CFX Manager software. Three biological replicates were performed.

## Supporting information

S1 Table Details of splicing variants of maize *GRAS* genes. (DOC)

S2 Table The maize GRAS genes in the PlantTFDB and PlnTFDB websites. (DOCX)

S3 Table Gene ID of *GRAS* gene family members from four model plants: *Arabidopsis thaliana, Medicago truncatula, Oryza sativa* and *Sorghum bicolor*. (DOCX)

S4 Table The structural features of motif 1–20. (DOC)

S5 Table Partial of the different cis-regulate elements in the 2000bp promoter region of the gene *ZmGRAS40* and *ZmGRAS62*. (DOCX)

S6 Table Primers used in the Real-time quantitative RT-PCR. (DOCX)

S1 Fig C-terminal conserved domains of maize *GRAS* genes. (TIF)

S2 Fig Three N-terminal specific motifs of LISCL subfamily. (TIF)
