# Supplementary material for: Identification and expression of GRAS family genes in maize (Zea mays L.)
Source: PLoS One. 2017 Sep 28;12(9):e0185418. doi: 10.1371/journal.pone.0185418 (PMC5619761; doi:10.1371/journal.pone.0185418)
Supplement: S1 Table — (DOC) [file pone.0185418.s001.doc]

**S1 Table. Details of splicing variants of maize GRAS genes.**

| **Name** | **ID** | **Splice variants** | **Exons** | **Coding exons** | **Transcript length** | **Translation length** |
| --- | --- | --- | --- | --- | --- | --- |
| ZmGRAS74 | GRMZM2G408012 | 2 | 2 | 2 | 932 | 179 |
|  |  |  | 3 | 3 | 756 | 251 |
| ZmGRAS62 | GRMZM2G173429 | 2 | 1 | 1 | 2287 | 623 |
|  |  |  | 2 | 1 | 1926 | 397 |
| ZmGRAS18 | GRMZM2G049159 | 3 | 3 | 1 | 3234 | 734 |
|  |  |  | 4 | 1 | 2886 | 734 |
|  |  |  | 2 | 2 | 887 | 89 |
| ZmGRAS54（D8） | GRMZM2G144744 | 3 | 1 | 1 | 3068 | 630 |
|  |  |  | 2 | 2 | 2238 | 584 |
|  |  |  | 2 | 2 | 827 | 232 |
| ZmGRAS59 | GRMZM2G163427 | 2 | 2 | 1 | 2578 | 546 |
|  |  |  | 1 | 1 | 2521 | 765 |
| ZmGRAS51 | GRMZM2G140085 | 2 | 1 | 1 | 1262 | 369 |
|  |  |  | 2 | 2 | 1138 | 320 |
| ZmGRAS48（SCR） | GRMZM2G131516 | 2 | 2 | 2 | 2449 | 668 |
|  |  |  | 1 | 1 | 868 | 140 |
| ZmGRAS35 | GRMZM2G098800 | 2 | 1 | 1 | 2783 | 708 |
|  |  |  | 2 | 2 | 2667 | 479 |
| ZmGRAS8 | GRMZM2G015080 | 2 | 2 | 2 | 2727 | 678 |
|  |  |  | 2 | 2 | 2099 | 530 |
| ZmGRAS80 | GRMZM5G825321 | 2 | 1 | 1 | 2889 | 721 |
|  |  |  | 2 | 2 | 1936 | 485 |
| ZmGRAS40 | GRMZM2G109869 | 2 | 1 | 1 | 2333 | 623 |
|  |  |  | 2 | 2 | 1646 | 429 |
| ZmGRAS13 | GRMZM2G028039 | 2 | 3 | 1 | 2421 | 545 |
|  |  |  | 2 | 2 | 653 | 98 |
| ZmGRAS43 | GRMZM2G114680 | 3 | 4 | 1 | 2181 | 467 |
|  |  |  | 1 | 1 | 1786 | 467 |
|  |  |  | 2 | 2 | 512 | 170 |
| ZmGRAS75 | GRMZM2G418899 | 2 | 5 | 4 | 1355 | 397 |
|  |  |  | 5 | 5 | 1315 | 431 |
| ZmGRAS33 | GRMZM2G098517 | 3 | 3 | 1 | 2380 | 558 |
|  |  |  | 2 | 1 | 2311 | 558 |
|  |  |  | 1 | 1 | 807 | 67 |
| ZmGRAS57 | GRMZM2G157679 | 2 | 1 | 1 | 3170 | 809 |
|  |  |  | 3 | 2 | 2722 | 812 |
| ZmGRAS27 | GRMZM2G082387 | 2 | 1 | 1 | 2156 | 447 |
|  |  |  | 2 | 2 | 1873 | 466 |
| ZmGRAS58 | GRMZM2G159475 | 4 | 1 | 1 | 2761 | 710 |
|  |  |  | 2 | 1 | 1354 | 197 |
|  |  |  | 2 | 1 | 1099 | 197 |
|  |  |  | 2 | 1 | 1088 | 197 |
