# Supplementary material for: Identification and expression of GRAS family genes in maize (Zea mays L.)
Source: PLoS One. 2017 Sep 28;12(9):e0185418. doi: 10.1371/journal.pone.0185418 (PMC5619761; doi:10.1371/journal.pone.0185418)
Supplement: S2 Table — (DOCX) [file pone.0185418.s002.docx]

**S2 Table. The maize GRAS genes in the PlantTFDB and PlnTFDB websites.**

| **The 104 maize GRAS genes in the PlantTFDB website** | **The 112 maize GRAS genes in the PlnTFDB website** |
| --- | --- |
| AC198366.3_FGP004 | AC187209.5_FGP031 |
| AC200124.3_FGP005 | AC191401.3_FGP045 |
| AC204621.4_FGP006 | AC198366.3_FGP040 |
| AC234164.1_FGP004 | AC198420.4_FGP056 |
| GRMZM2G001426_P01 | AC207334.3_FGP042 |
| GRMZM2G011947_P01 | AC217624.1_FGP038 |
| GRMZM2G013016_P01 | AC217999.3_FGP030 |
| GRMZM2G015080_P01 | GRMZM2G001426_P01 |
| GRMZM2G015080_P02 | GRMZM2G013016_P01 |
| GRMZM2G018254_P01 | GRMZM2G015004_P01 |
| GRMZM2G019060_P01 | GRMZM2G015004_P03 |
| GRMZM2G023872_P01 | GRMZM2G015080_P01 |
| GRMZM2G024973_P01 | GRMZM2G015080_P02 |
| GRMZM2G028039_P02 | GRMZM2G015696_P01 |
| GRMZM2G028438_P01 | GRMZM2G018254_P01 |
| GRMZM2G028608_P01 | GRMZM2G019060_P01 |
| GRMZM2G037286_P01 | GRMZM2G022717_P01 |
| GRMZM2G037792_P01 | GRMZM2G023872_P01 |
| GRMZM2G049159_P01 | GRMZM2G024320_P01 |
| GRMZM2G049159_P03 | GRMZM2G024320_P02 |
| GRMZM2G051785_P01 | GRMZM2G024973_P01 |
| GRMZM2G055263_P01 | GRMZM2G028039_P02 |
| GRMZM2G060265_P01 | GRMZM2G028438_P01 |
| GRMZM2G070371_P01 | GRMZM2G028608_P01 |
| GRMZM2G073779_P01 | GRMZM2G037286_P01 |
| GRMZM2G073805_P01 | GRMZM2G037792_P01 |
| GRMZM2G073823_P01 | GRMZM2G038199_P01 |
| GRMZM2G079470_P01 | GRMZM2G043233_P01 |
| GRMZM2G082387_P01 | GRMZM2G049159_P01 |
| GRMZM2G089636_P01 | GRMZM2G049159_P03 |
| GRMZM2G089662_P01 | GRMZM2G051785_P01 |
| GRMZM2G089782_P01 | GRMZM2G055263_P01 |
| GRMZM2G091656_P01 | GRMZM2G057736_P01 |
| GRMZM2G097456_P01 | GRMZM2G060265_P01 |
| GRMZM2G098517_P01 | GRMZM2G067446_P01 |
| GRMZM2G098517_P02 | GRMZM2G068223_P01 |
| GRMZM2G098784_P01 | GRMZM2G070371_P01 |
| GRMZM2G098800_P01 | GRMZM2G071183_P01 |
| GRMZM2G098800_P02 | GRMZM2G072576_P01 |
| GRMZM2G104342_P01 | GRMZM2G073741_P01 |
| GRMZM2G106336_P01 | GRMZM2G073779_P01 |
| GRMZM2G106356_P01 | GRMZM2G073805_P01 |
| GRMZM2G106548_P01 | GRMZM2G073823_P01 |
| GRMZM2G109869_P01 | GRMZM2G076855_P01 |
| GRMZM2G109869_P02 | GRMZM2G079470_P01 |
| GRMZM2G110067_P01 | GRMZM2G079980_P01 |
| GRMZM2G110579_P01 | GRMZM2G082387_P01 |
| GRMZM2G114680_P01 | GRMZM2G082656_P01 |
| GRMZM2G114680_P02 | GRMZM2G089636_P01 |
| GRMZM2G114680_P03 | GRMZM2G089662_P01 |
| GRMZM2G116638_P01 | GRMZM2G091656_P01 |
| GRMZM2G117949_P01 | GRMZM2G096928_P01 |
| GRMZM2G125501_P01 | GRMZM2G097456_P01 |
| GRMZM2G129154_P01 | GRMZM2G098517_P01 |
| GRMZM2G131516_P01 | GRMZM2G098517_P02 |
| GRMZM2G131516_P02 | GRMZM2G098784_P01 |
| GRMZM2G132794_P01 | GRMZM2G098800_P01 |
| GRMZM2G133169_P01 | GRMZM2G098800_P02 |
| GRMZM2G140085_P01 | GRMZM2G104342_P01 |
| GRMZM2G140085_P02 | GRMZM2G106336_P01 |
| GRMZM2G140094_P01 | GRMZM2G106356_P01 |
| GRMZM2G143433_P01 | GRMZM2G106548_P01 |
| GRMZM2G144744_P01 | GRMZM2G109869_P01 |
| GRMZM2G144744_P02 | GRMZM2G110067_P01 |
| GRMZM2G146018_P01 | GRMZM2G114680_P01 |
| GRMZM2G153333_P01 | GRMZM2G114680_P02 |
| GRMZM2G157679_P01 | GRMZM2G116638_P01 |
| GRMZM2G157679_P02 | GRMZM2G117421_P01 |
| GRMZM2G159475_P01 | GRMZM2G117426_P01 |
| GRMZM2G159475_P02 | GRMZM2G120504_P01 |
| GRMZM2G159475_P03 | GRMZM2G122312_P01 |
| GRMZM2G159475_P04 | GRMZM2G122741_P01 |
| GRMZM2G163427_P01 | GRMZM2G124256_P01 |
| GRMZM2G163427_P02 | GRMZM2G125501_P01 |
| GRMZM2G169636_P01 | GRMZM2G128893_P01 |
| GRMZM2G172657_P01 | GRMZM2G129154_P01 |
| GRMZM2G173429_P01 | GRMZM2G130188_P01 |
| GRMZM2G173429_P02 | GRMZM2G130825_P01 |
| GRMZM2G176537_P01 | GRMZM2G130825_P02 |
| GRMZM2G179325_P01 | GRMZM2G131516_P01 |
| GRMZM2G313078_P01 | GRMZM2G132794_P01 |
| GRMZM2G317287_P01 | GRMZM2G133169_P01 |
| GRMZM2G335814_P01 | GRMZM2G140085_P01 |
| GRMZM2G342217_P01 | GRMZM2G140094_P01 |
| GRMZM2G346706_P01 | GRMZM2G143433_P01 |
| GRMZM2G348780_P01 | GRMZM2G144744_P01 |
| GRMZM2G359304_P01 | GRMZM2G144744_P02 |
| GRMZM2G368909_P01 | GRMZM2G146018_P01 |
| GRMZM2G386362_P01 | GRMZM2G146938_P01 |
| GRMZM2G408012_P01 | GRMZM2G146938_P02 |
| GRMZM2G418899_P01 | GRMZM2G147945_P01 |
| GRMZM2G418899_P02 | GRMZM2G148759_P01 |
| GRMZM2G420280_P01 | GRMZM2G153333_P01 |
| GRMZM2G425366_P01 | GRMZM2G157679_P01 |
| GRMZM2G431309_P01 | GRMZM2G157679_P02 |
| GRMZM5G821439_P01 | GRMZM2G159475_P01 |
| GRMZM5G825321_P01 | GRMZM2G159475_P02 |
| GRMZM5G825321_P02 | GRMZM2G159475_P03 |
| GRMZM5G826526_P01 | GRMZM2G159475_P04 |
| GRMZM5G868355_P01 | GRMZM2G159980_P01 |
| GRMZM5G874545_P01 | GRMZM2G159980_P02 |
| GRMZM5G885274_P01 | GRMZM2G163427_P01 |
| GRMZM5G889326_P01 | GRMZM2G163427_P02 |
| GRMZM5G895672_P01 | GRMZM2G167471_P01 |
|  | GRMZM2G167493_P01 |
|  | GRMZM2G169378_P01 |
|  | GRMZM2G169636_P01 |
|  | GRMZM2G172657_P01 |
|  | GRMZM2G173429_P01 |
|  | GRMZM2G173429_P02 |
|  | GRMZM2G176537_P01 |
|  | GRMZM2G179325_P01 |

Genes written in the red words were different genes between the PlantTFDB and PlnTFDB websites.
