# Supplementary material for: Identification and expression of GRAS family genes in maize (Zea mays L.)
Source: PLoS One. 2017 Sep 28;12(9):e0185418. doi: 10.1371/journal.pone.0185418 (PMC5619761; doi:10.1371/journal.pone.0185418)
Supplement: S3 Table — (DOCX) [file pone.0185418.s003.docx]

**S3 Table. Gene ID of GRAS gene family members from four model plants: *Arabidopsis thaliana，Medicago truncatula ，Oryza sativa* and *Sorghum b*icolo*r.***

| ***Arabidopsis thaliana*** | | ***Medicago truncatula*** | | ***Oryza sativa*** | | ***Sorghum bicolor*** | |
| --- | --- | --- | --- | --- | --- | --- | --- |
| Name | Gene ID | Name | Gene ID | Name | Gene ID | Name | Gene ID |
| AtGRAS1 | AT1G07520 | MtGRAS1 | Medtr0092s0100 | OsGRAS1 | LOC_Os01g45860 | SbGRAS1 | Sobic.001G033200 |
| AtGRAS2 | AT1G07530 | MtGRAS2 | Medtr1g029420 | OsGRAS2 | LOC_Os01g62460 | SbGRAS2 | Sobic.001G109100 |
| AtGRAS3 | AT1G14920 | MtGRAS3 | Medtr1g069725 | OsGRAS3 | LOC_Os01g65900 | SbGRAS3 | Sobic.001G120900 |
| AtGRAS4 | AT1G21450 | MtGRAS4 | Medtr1g086970 | OsGRAS4 | LOC_Os01g67650 | SbGRAS4 | Sobic.001G174100 |
| AtGRAS5 | AT1G50420 | MtGRAS5 | Medtr1g096030 | OsGRAS5 | LOC_Os01g67670 | SbGRAS5 | Sobic.001G174200 |
| AtGRAS6 | AT1G50600 | MtGRAS6 | Medtr1g106590 | OsGRAS6 | LOC_Os01g71970 | SbGRAS6 | Sobic.001G174300 |
| AtGRAS7 | AT1G55580 | MtGRAS7 | Medtr2g026250 | OsGRAS7 | LOC_Os02g10360 | SbGRAS7 | Sobic.001G174500 |
| AtGRAS8 | AT1G63100 | MtGRAS8 | Medtr2g034250 | OsGRAS8 | LOC_Os02g21685 | SbGRAS8 | Sobic.001G181100 |
| AtGRAS9 | AT1G66350 | MtGRAS9 | Medtr2g034260 | OsGRAS9 | LOC_Os02g44360 | SbGRAS9 | Sobic.001G304500 |
| AtGRAS10 | AT2G01570 | MtGRAS10 | Medtr2g034280 | OsGRAS10 | LOC_Os02g44370 | SbGRAS10 | Sobic.001G327900 |
| AtGRAS11 | AT2G04890 | MtGRAS11 | Medtr2g082090 | OsGRAS11 | LOC_Os02g45760 | SbGRAS11 | Sobic.001G341400 |
| AtGRAS12 | AT2G29060 | MtGRAS12 | Medtr2g089100 | OsGRAS12 | LOC_Os03g09280 | SbGRAS12 | Sobic.001G421900 |
| AtGRAS13 | AT2G29065 | MtGRAS13 | Medtr2g097310 | OsGRAS13 | LOC_Os03g15680 | SbGRAS13 | Sobic.001G428600 |
| AtGRAS14 | AT2G37650 | MtGRAS14 | Medtr2g097350 | OsGRAS14 | LOC_Os03g29480 | SbGRAS14 | Sobic.001G472600 |
| AtGRAS15 | AT2G45160 | MtGRAS15 | Medtr2g097380 | OsGRAS15 | LOC_Os03g31880 | SbGRAS15 | Sobic.002G301800 |
| AtGRAS16 | AT3G03450 | MtGRAS16 | Medtr2g097390 | OsGRAS16 | LOC_Os03g37900 | SbGRAS16 | Sobic.002G316500 |
| AtGRAS17 | AT3G13840 | MtGRAS17 | Medtr2g097410 | OsGRAS17 | LOC_Os03g40080 | SbGRAS17 | Sobic.002G330300 |
| AtGRAS18 | AT3G46600 | MtGRAS18 | Medtr2g097463 | OsGRAS18 | LOC_Os03g48450 | SbGRAS18 | Sobic.002G342800 |
| AtGRAS19 | AT3G49950 | MtGRAS19 | Medtr2g097467 | OsGRAS19 | LOC_Os03g49990 | SbGRAS19 | Sobic.002G354900 |
| AtGRAS20 | AT3G50650 | MtGRAS20 | Medtr2g097473 | OsGRAS20 | LOC_Os03g51330 | SbGRAS20 | Sobic.002G357800 |
| AtGRAS21 | AT3G54220 | MtGRAS21 | Medtr2g099110 | OsGRAS21 | LOC_Os04g35250 | SbGRAS21 | Sobic.002G359800 |
| AtGRAS22 | AT3G60630 | MtGRAS22 | Medtr3g021320 | OsGRAS22 | LOC_Os04g37440 | SbGRAS22 | Sobic.002G372100 |
| AtGRAS23 | AT4G00150 | MtGRAS23 | Medtr3g022005 | OsGRAS23 | LOC_Os04g46860 | SbGRAS23 | Sobic.003G237201 |
| AtGRAS24 | AT4G08250 | MtGRAS24 | Medtr3g022580 | OsGRAS24 | LOC_Os04g49110 | SbGRAS24 | Sobic.003G352600 |
| AtGRAS25 | AT4G17230 | MtGRAS25 | Medtr3g022830 | OsGRAS25 | LOC_Os04g50060 | SbGRAS25 | Sobic.003G377900 |
| AtGRAS26 | AT4G36710 | MtGRAS26 | Medtr3g025340 | OsGRAS26 | LOC_Os05g31380 | SbGRAS26 | Sobic.003G386900 |
| AtGRAS27 | AT4G37650 | MtGRAS27 | Medtr3g027430 | OsGRAS27 | LOC_Os05g31420 | SbGRAS27 | Sobic.003G392800 |
| AtGRAS28 | AT5G17490 | MtGRAS28 | Medtr3g053270 | OsGRAS28 | LOC_Os05g40710 | SbGRAS28 | Sobic.003G423900 |
| AtGRAS29 | AT5G41920 | MtGRAS29 | Medtr3g056110 | OsGRAS29 | LOC_Os05g42130 | SbGRAS29 | Sobic.004G080300 |
| AtGRAS30 | AT5G48150 | MtGRAS30 | Medtr3g065980 | OsGRAS30 | LOC_Os05g49930 | SbGRAS30 | Sobic.004G281200 |
| AtGRAS31 | AT5G52510 | MtGRAS31 | Medtr3g072710 | OsGRAS31 | LOC_Os06g01620 | SbGRAS31 | Sobic.004G290800 |
| AtGRAS32 | AT5G59450 | MtGRAS32 | Medtr3g089055 | OsGRAS32 | LOC_Os06g03710 | SbGRAS32 | Sobic.004G291000 |
| AtGRAS33 | AT5G66770 | MtGRAS33 | Medtr4g026485 | OsGRAS33 | LOC_Os06g10900 | SbGRAS33 | Sobic.005G017500 |
| AtGRAS34 | AT5G67411 | MtGRAS34 | Medtr4g064120 | OsGRAS34 | LOC_Os06g40780 | SbGRAS34 | Sobic.005G029600 |
|  |  | MtGRAS35 | Medtr4g064150 | OsGRAS35 | LOC_Os07g16330 | SbGRAS35 | Sobic.005G046250 |
|  |  | MtGRAS36 | Medtr4g064160 | OsGRAS36 | LOC_Os07g36170 | SbGRAS36 | Sobic.005G123000 |
|  |  | MtGRAS37 | Medtr4g064180 | OsGRAS37 | LOC_Os07g38030 | SbGRAS37 | Sobic.005G208950 |
|  |  | MtGRAS38 | Medtr4g064200 | OsGRAS38 | LOC_Os07g39470 | SbGRAS38 | Sobic.005G209000 |
|  |  | MtGRAS39 | Medtr4g074310 | OsGRAS39 | LOC_Os07g39820 | SbGRAS39 | Sobic.005G209100 |
|  |  | MtGRAS40 | Medtr4g074320 | OsGRAS40 | LOC_Os07g40020 | SbGRAS40 | Sobic.005G209200 |
|  |  | MtGRAS41 | Medtr4g076020 | OsGRAS41 | LOC_Os10g22430 | SbGRAS41 | Sobic.005G229400 |
|  |  | MtGRAS42 | Medtr4g076140 | OsGRAS42 | LOC_Os10g40390 | SbGRAS42 | Sobic.005G229500 |
|  |  | MtGRAS43 | Medtr4g077760 | OsGRAS43 | LOC_Os11g03110 | SbGRAS43 | Sobic.005G229600 |
|  |  | MtGRAS44 | Medtr4g095500 | OsGRAS44 | LOC_Os11g04400 | SbGRAS44 | Sobic.005G229700 |
|  |  | MtGRAS45 | Medtr4g097080 | OsGRAS45 | LOC_Os11g04570 | SbGRAS45 | Sobic.005G229900 |
|  |  | MtGRAS46 | Medtr4g102790 | OsGRAS46 | LOC_Os11g04590 | SbGRAS46 | Sobic.005G230000 |
|  |  | MtGRAS47 | Medtr4g104020 | OsGRAS47 | LOC_Os11g06180 | SbGRAS47 | Sobic.005G230100 |
|  |  | MtGRAS48 | Medtr4g122240 | OsGRAS48 | LOC_Os11g11600 | SbGRAS48 | Sobic.005G230200 |
|  |  | MtGRAS49 | Medtr4g133660 | OsGRAS49 | LOC_Os11g31100 | SbGRAS49 | Sobic.005G230300 |
|  |  | MtGRAS50 | Medtr5g009080 | OsGRAS50 | LOC_Os11g47870 | SbGRAS50 | Sobic.005G230400 |
|  |  | MtGRAS51 | Medtr5g015490 | OsGRAS51 | LOC_Os11g47890 | SbGRAS51 | Sobic.005G230500 |
|  |  | MtGRAS52 | Medtr5g015950 | OsGRAS52 | LOC_Os11g47900 | SbGRAS52 | Sobic.005G230600 |
|  |  | MtGRAS53 | Medtr5g019750 | OsGRAS53 | LOC_Os11g47910 | SbGRAS53 | Sobic.005G230700 |
|  |  | MtGRAS54 | Medtr5g058860 | OsGRAS54 | LOC_Os11g47920 | SbGRAS54 | Sobic.005G230800 |
|  |  | MtGRAS55 | Medtr5g094450 | OsGRAS55 | LOC_Os12g02870 | SbGRAS55 | Sobic.005G230900 |
|  |  | MtGRAS56 | Medtr5g097480 | OsGRAS56 | LOC_Os12g04200 | SbGRAS56 | Sobic.005G231000 |
|  |  | MtGRAS57 | Medtr6g047750 | OsGRAS57 | LOC_Os12g04370 | SbGRAS57 | Sobic.006G083201 |
|  |  | MtGRAS58 | Medtr7g027190 | OsGRAS58 | LOC_Os12g04380 | SbGRAS58 | Sobic.006G093700 |
|  |  | MtGRAS59 | Medtr7g057230 | OsGRAS59 | LOC_Os12g06540 | SbGRAS59 | Sobic.006G093800 |
|  |  | MtGRAS60 | Medtr7g062120 | OsGRAS60 | LOC_Os12g38490 | SbGRAS60 | Sobic.006G172900 |
|  |  | MtGRAS61 | Medtr7g069740 |  |  | SbGRAS61 | Sobic.006G188900 |
|  |  | MtGRAS62 | Medtr7g074650 |  |  | SbGRAS62 | Sobic.008G012700 |
|  |  | MtGRAS63 | Medtr7g109580 |  |  | SbGRAS63 | Sobic.008G012800 |
|  |  | MtGRAS64 | Medtr8g020840 |  |  | SbGRAS64 | Sobic.008G023401 |
|  |  | MtGRAS65 | Medtr8g077940 |  |  | SbGRAS65 | Sobic.008G046700 |
|  |  | MtGRAS66 | Medtr8g093070 |  |  | SbGRAS66 | Sobic.008G138600 |
|  |  | MtGRAS67 | Medtr8g442410 |  |  | SbGRAS67 | Sobic.008G168400 |
|  |  |  |  |  |  | SbGRAS68 | Sobic.009G011000 |
|  |  |  |  |  |  | SbGRAS69 | Sobic.009G011100 |
|  |  |  |  |  |  | SbGRAS70 | Sobic.009G011200 |
|  |  |  |  |  |  | SbGRAS71 | Sobic.009G011300 |
|  |  |  |  |  |  | SbGRAS72 | Sobic.009G117700 |
|  |  |  |  |  |  | SbGRAS73 | Sobic.009G117800 |
|  |  |  |  |  |  | SbGRAS74 | Sobic.009G143400 |
|  |  |  |  |  |  | SbGRAS75 | Sobic.009G177100 |
|  |  |  |  |  |  | SbGRAS76 | Sobic.009G187500 |
|  |  |  |  |  |  | SbGRAS77 | Sobic.010G003700 |
|  |  |  |  |  |  | SbGRAS78 | Sobic.010G017400 |
|  |  |  |  |  |  | SbGRAS79 | Sobic.010G115450 |
|  |  |  |  |  |  | SbGRAS80 | Sobic.010G189500 |
