# Supplementary material for: Identification and expression of GRAS family genes in maize (Zea mays L.)
Source: PLoS One. 2017 Sep 28;12(9):e0185418. doi: 10.1371/journal.pone.0185418 (PMC5619761; doi:10.1371/journal.pone.0185418)
Supplement: S4 Table — (DOC) [file pone.0185418.s004.doc]

**S4 Table. The structural features of motif 1-20.**

| **Name** | **Amino acid sequence** |
| --- | --- |
| Motif 1 | N[VI][VI]ACEG[AS][DE]RVER[PH]ET[YFL][GKR][QK]W[QR]AR[MN]xRAG[FL] |
| Motif 2 | [VIL]H[IV][VI]D[FLY][GD]IxQGFQW[PA][ST]L[LI]QAL |
| Motif 3 | NA[PA][PF]FLTRFREALF[YH]YSALFD[SM]L[DE]A[TA]LPR |
| Motif 4 | EDGGWLL[LQ]GWKGRPL[YV][AS][VA]S[AT]W |
| Motif 5 | YQL[FLY]Y[EDA][AL][CS]PFL[KR][FV]AHF[TF][AS]NQ[AT]IL[ED]AVAGE |
| Motif 6 | xGD[AP]TQRLA[AH][YC]FAE[AG]L[EA]ARLAG[TS]GSQLYR |
| Motif 7 | [SA][KR]WE[AE][VIL]R[AV]EDLN[IV][DE]P[DG]E[AV][LV][AVI]VN[CFS]L[FLY]Q[LF]H[NH]L[PM]DE[ST]V |
| Motif 8 | R[PE]GGPPE[LV]R[IL]TG[IV][DG]DPQ[PS][GA]F[RA]P[AT]G |
| Motif 9 | [QT]LL[LI][HA]CAQAV[AE]A[GDN][DN]RxAAxELL[AK]Q[IL]RQ[HL][AS] |
| Motif 10 | [DS]IP[SN]PRDRVL[RN][NT][VI][RK]K[ML][RN]PDV[FV][IT][LH][VG][EIV][QV][NE][GA][SN] |
| Motif 11 | EE[TV]GRRL[AS]D[FY]ARS[LF]GVP |
| Motif 12 | [FLV]LRL[VL][RK]RLAPK[VLI]V[VT][VL]V[ED][QE][EDG]A[DG] |
| Motif 13 | [DE][SD]AER[LA]L[VI]E[RQ]xL[LF][GA]R |
| Motif 14 | PVPLSNx[AV][EV][AS]QA[RKD]LLL[KR]RYxx |
| Motif 15 | DM[VT]LPYISRMLME[ED]D[ID]D[DE]K[FV]S |
| Motif 16 | [GEN]G[AS][DN]M[DS][ML]L[NSTA][MLS][AQ]FL[KR]GMEEA[NK]K[FL]LP[TV]x[DNS][KSN]L |
| Motif 17 | HPALL[QA][AV][QE][QK]PF[AY][DQ]IL |
| Motif 18 | K[DEK]xYH[KR]DF[VL]ID |
| Motif 19 | QLPL[DN]P[DES][IV][VM]K[VA][VAL][RK]D[KE] |
| Motif 20 | [FL][QE]F[AST]A[IY][NS][LAV]DA[LFI][DN]PAE[LF][VI][AS][IAP][TS][AS][DG][DE][AV]VAV[HCV]LPVG |
