# Supplementary material for: Identification and expression of GRAS family genes in maize (Zea mays L.)
Source: PLoS One. 2017 Sep 28;12(9):e0185418. doi: 10.1371/journal.pone.0185418 (PMC5619761; doi:10.1371/journal.pone.0185418)
Supplement: S5 Table — (DOCX) [file pone.0185418.s005.docx]

**S5 Table. Partial of the different cis-regulate elements in the 2000bp promoter region of the gene  *ZmGRAS40* and *ZmGRAS62*.**

| **Site name** | **Sequence** | **position** | | **Function** |
| --- | --- | --- | --- | --- |
|  |  | ***ZmGRAS40*** | ***ZmGRAS62*** |  |
| CPBCSPOR | TATTAG | A(-1673)/S(-1902) | - | Cytokinin-enhanced Protein Binding site |
| BOXIINTPATPB | ATAGAA | A(-1916) | - | activity of plastid atpB gene in the tobacco |
| -10PEHVPSBD | TATTCT | A(-1864) | - | Involved in the expression of the plastid gene psbD in the barley |
| GARE2OSREP1 | TAACGTA | - | A(-1506) | Gibberellin-responsive element |
| GAREAT | TAACAAR | - | S(-1600)/A(-1998) | GARE (GA-responsive element) |
| MYBGAHV | TAACAAA | - | S(-1600)/A(-1998) | Central element of gibberellin (GA) response complex (GARC) |
| MYBPLANT | WACCWAMC | - | S(-387)/A(-495)/  S(-1489)/A(1662) | Plant MYB binding site, regulating phenylpropanoid and lignin biosynthesis in tobacco |
| XYLAT | ACAAAGAA | - | S(-1699) | cis-element identified among the promoters of the "core xylem |

S, the sense strand; A, the antisense strand; W: A/T; M: A/C; -1: the first base upstream of the start codon.
