# Supplementary material for: Identification and expression of GRAS family genes in maize (Zea mays L.)
Source: PLoS One. 2017 Sep 28;12(9):e0185418. doi: 10.1371/journal.pone.0185418 (PMC5619761; doi:10.1371/journal.pone.0185418)
Supplement: S6 Table — (DOCX) [file pone.0185418.s006.docx]

**S6 Table. Primers used in the Real-time quantitative RT-PCR.**

| **Primer name** | **Sequence** |
| --- | --- |
| ZmPL067F-actin | GGCTGGGATGAGAATGAAGA |
| ZmPL067R-actin | TTCTGCCTTACACAATCAGCA |
| ZmGRAS10-66bp.F | CGTGCATGTATGTATGGCGAGTTC |
| ZmGRAS10-66bp.R | ACGCGTGAGCCATTCAGCTTTC |
| ZmGRAS12-78bp.F | AGGACGAACCGGATTTCGTTAGC |
| ZmGRAS12-78bp.R | TGTGAGCATAGCCAAGGTTCGG |
| ZmGRAS19-63bp.F | TGAGGAACGATTGGAAGCCACAG |
| ZmGRAS19-63bp.R | AGGAATATGGGCATGGCAAATGGG |
| ZmGRAS25-73bp.F | TGGAAGGGACGTGTTCTCTATGGC |
| ZmGRAS25-73bp.R | AGAGGGCCTTTAGTCATGGGTCAG |
| ZmGRAS31-60bp.F | TGCTGGTTGGGTTGTTCAGCAG |
| ZmGRAS31-60bp.R | ACTGAAGCCTGCCTTTCTCATGC |
| ZmGRAS40-62bp.F | TGGGTGAGGAGTGATGAGTTTGC |
| ZmGRAS40-62bp.R | TGAGTCGCTACAGCTAGGAAGG |
| ZmGRAS54-72bp.F | GATCGGCCTGTGTTCGTTCTTG |
| ZmGRAS54-72bp.R | ACCATACTTGACGGGATCGAAGC |
| ZmGRAS61-76bp.F | TGTGTATGGCGAGCTGAATGGC |
| ZmGRAS61-76bp.R | TGTGTGCGTGAACTAGCAATCCC |
| ZmGRAS62-64bp.F | TGGGTGAGGAGTGATGAGTTTGTC |
| ZmGRAS62-64bp.R | ATGAGCCGCTACCAACTAGGAAGG |
| ZmGRAS67-79bp.F | TCGCATGTGAAGGATCCGAAAGG |
| ZmGRAS67-79bp.R | AGGCCAGCATTCAGGTTTCGTG |
| ZmGRAS69-77bp.F | AGCATGTGAAGGTGCAGAGAGG |
| ZmGRAS69-77bp.R | ACCCAGCCTTGAGGATTCTTGC |
