# Supplementary figures and images for: Identification and expression of GRAS family genes in maize (Zea mays L.)
Source: PLoS One. 2017 Sep 28;12(9):e0185418. doi: 10.1371/journal.pone.0185418 (PMC5619761; doi:10.1371/journal.pone.0185418)

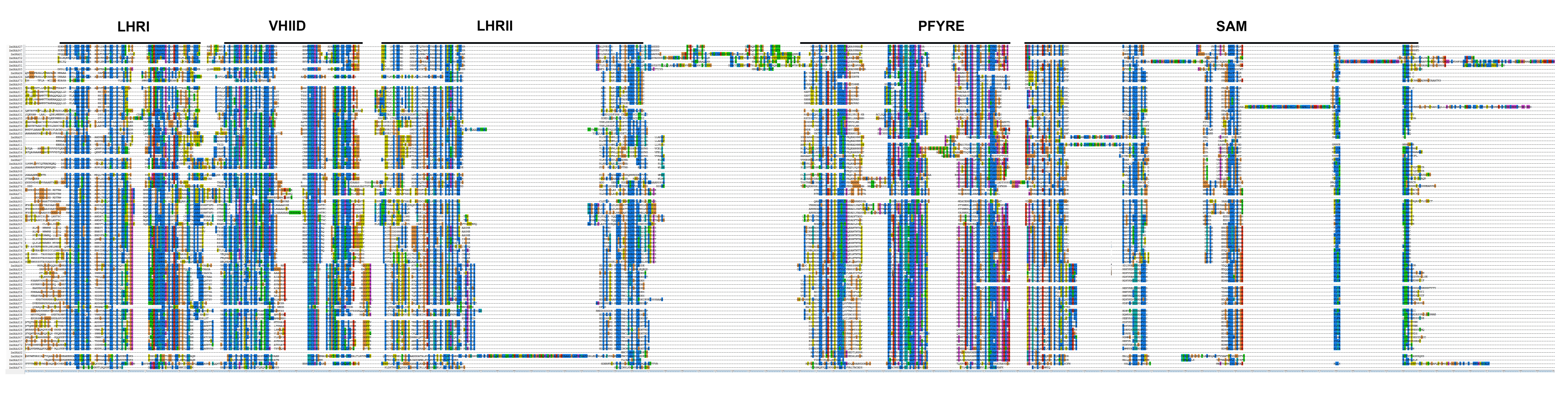

Supplement: S1 Fig — (TIF) [file pone.0185418.s007.tif]

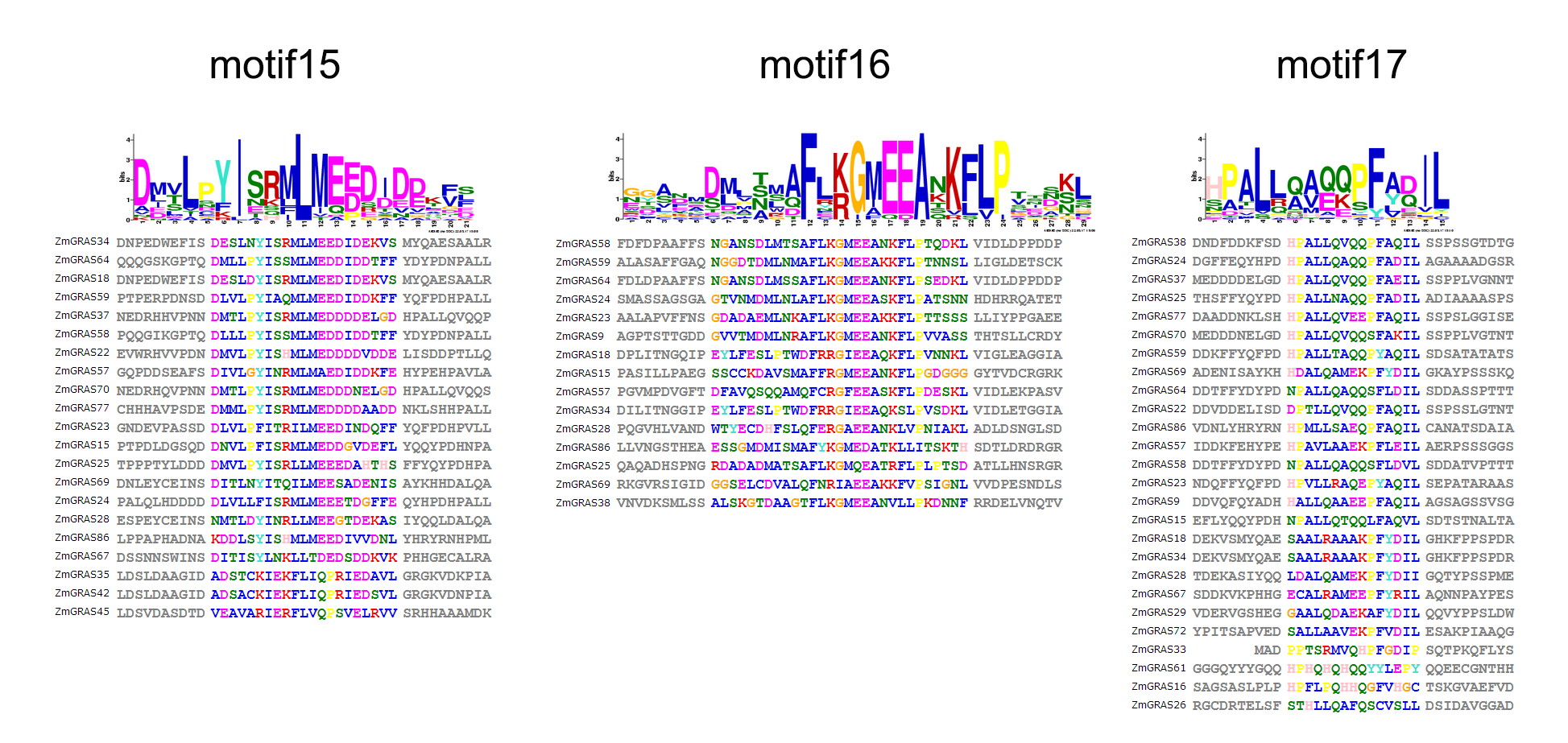

Supplement: S2 Fig — (TIF) [file pone.0185418.s008.tif]
